# The clinical spectrum of severe childhood malaria in Eastern Uganda

**DOI:** 10.1186/s12936-020-03390-7

**Published:** 2020-09-03

**Authors:** Peter Olupot-Olupot, Charles Engoru, Julius Nteziyaremye, Martin Chebet, Tonny Ssenyondo, Rita Muhindo, Gideon Nyutu, Alexander W. Macharia, Sophie Uyoga, Carolyne M. Ndila, Charles Karamagi, Kathryn Maitland, Thomas N. Williams

**Affiliations:** 1 Faculty of Health Sciences, Busitema University, Mbale Campus, P.O. Box 1460, Mbale, Uganda; 2 Mbale Clinical Research Institute, P.O. Box 1966, Mbale, Uganda; 3 Soroti Regional Referral Hospital, P.O. Box 289, Soroti, Uganda; 4 KEMRI-Wellcome Trust Research Programme, Centre for Geographic Medicine Research-Coast, Kilifi, Kenya; 5 Makerere University College of Health Sciences, P.O. Box 7072, Kampala, Uganda; 6 Institute of Global Health Innovation, Imperial College, Medical School Building St Mary’s Campus, Imperial College, London, W2 1PG UK

**Keywords:** Severe malaria, Severe anaemia, Dark red or black urine, *P. falciparum* malaria, Children, Uganda

## Abstract

**Background:**

Few recent descriptions of severe childhood malaria have been published from high-transmission regions. In the current study, the clinical epidemiology of severe malaria in Mbale, Eastern Uganda, is described, where the entomological inoculation rate exceeds 100 infective bites per year.

**Methods:**

A prospective descriptive study was conducted to determine the prevalence, clinical spectrum and outcome of severe *Plasmodium falciparum* malaria at Mbale Regional Referral Hospital in Eastern Uganda. All children aged 2 months–12 years who presented on Mondays to Fridays between 8.00 am and 5.00 pm from 5th May 2011 until 30th April 2012 were screened for parasitaemia. Clinical and laboratory data were then collected from all *P. falciparum* positive children with features of WHO-defined severe malaria by use of a standardized proforma.

**Results:**

A total of 10 208 children were screened of which 6582 (64%) had a positive blood film. Of these children, 662 (10%) had clinical features of severe malaria and were consented for the current study. Respiratory distress was the most common severity feature (554; 83.7%), while 365/585 (62.4%) had hyperparasitaemia, 177/662 (26.7%) had clinical jaundice, 169 (25.5%) had severe anaemia, 134/660 (20.2%) had hyperlactataemia (lactate ≥ 5 mmol/L), 93 (14.0%) had passed dark red or black urine, 52 (7.9%) had impaired consciousness and 49/662 (7.4%) had hypoxaemia (oxygen saturations < 90%). In-hospital mortality was 63/662 (9.5%) overall but was higher in children with either cerebral malaria (33.3%) or severe anaemia (19.5%). Factors that were independently associated with mortality on multivariate analysis included severe anaemia [odds ratio (OR) 5.36; 2.16–1.32; *P *= 0.0002], hyperlactataemia (OR 3.66; 1.72–7.80; *P *= 0.001), hypoxaemia (OR) 3.64 (95% CI 1.39–9.52; *P *= 0.008), and hepatomegaly (OR 2.29; 1.29–4.06; *P *= 0.004). No independent association was found between mortality and either coma or hyperparasitaemia.

**Conclusions:**

Severe childhood malaria remains common in Eastern Uganda where it continues to be associated with high mortality. An unusually high proportion of children with severe malaria had jaundice or gave a history of having recently passed dark red or black urine, an issue worthy of further investigation.

## Background

Despite the recent control strategies of national and international communities, *Plasmodium falciparum* malaria remains a major cause of morbidity and mortality in tropical countries, especially within the WHO Africa Region which accounts for the majority of cases worldwide [1]. According to the latest World Health Organization (WHO) report, six countries accounted for more than half of all malaria cases worldwide in 2018: Nigeria (25%), the Democratic Republic of the Congo (12%), Uganda (5%), and Côte d’Ivoire, Mozambique and Niger (4% each) [1].

In Uganda, the most recent descriptive studies of severe malaria, conducted in the central, south-western and south-eastern parts of the country, were published more than 15 years ago [2, 3]. Whereas the clinical spectrum of severe malaria that they described was broadly similar to that in previous studies [4, 5], the picture from the western part of the country was more consistent with descriptions from low transmission settings [6]. In recent years, a growing frequency of blackwater fever, a syndrome characterized by the triad of anaemia, jaundice and the passage of dark red or black urine, often associated with malaria parasitaemia [7], has been observed among children presenting to our hospital in Mbale in Eastern Uganda, a complication that has not featured strongly in previous descriptions of severe childhood malaria from Africa. The current study was conducted with the aim of providing a contemporary description of the clinical spectrum of severe childhood malaria in Eastern Uganda, and to identify the factors associated with mortality.

## Methods

### Clinical and laboratory data

We conducted a prospective descriptive study within the Paediatric Acute Care Unit (PACU) of Mbale Regional Referral Hospital (MRRH) between 5^th^ May 2011 and 30th April 2012. MRRH is a 470-bed hospital with 95 paediatric beds that is situated in a region of Eastern Uganda with hyper-endemic malaria transmission [8]. All children presenting to MRRH with acute illnesses are admitted to the PACU for 24 h, which generally receives approximately 17 000 admissions per year. All children aged 2 months to 12 years who were admitted to PACU between 8:00 am and 5:00 pm from Mondays to Fridays were screened for malaria, and all those who had both a positive *P. falciparum* blood film and one or more clinical or laboratory features of WHO-defined severe malaria were recruited [6, 9–12] (Table 1).

**Table 1:** Criteria used as indicators of severe malaria in the study

Clinical criteria	Definition
Clinical jaundice	Yellow mucous membranes noted in sufficient daylight
Respiratory distress	Increased work of breathing, manifesting as deep, fast or very slow breathing, including retractions and the use of accessory muscles
Severe anaemia	Haemoglobin < 5 g/dL
Prostration	Generalized weakness so that the patient is unable walk or sit up without assistance
Coma	Unrousable state with a corresponding Blantyre Coma Score (BCS) of ≤ 2 for which no other cause other than malaria could be identified
Haemoglobinuria	History of or clinician-observed red or cola-coloured urine
Multiple convulsions	More than two grand-mal seizures during the 24-h period preceding admission
Spontaneous bleeding	Physically un-induced and irrepressible bleeding from at least 2 non-traumatized sites in a patient with severe malaria without previous history of abnormal bleeding
Laboratory criteria	
Hyperlactataemia	Lactate > 5 mmol/L
Hyperparasitaemia	>5% parasitized erythrocytes or > 250 000 parasites/μL
Hyperpyrexia	Axillary temperature ≥ 40.0^°^C
Hypoxaemia	Oxygen saturation < 90%
Hypoglycaemia	Whole blood glucose concentration < 2.2 mmol/L
Metabolic acidosis	Plasma bicarbonate < 15 mmol/L

Standard case report forms (CRFs) were used to collect data on clinical, laboratory and socio-demographic factors, along with subsequent treatment and outcome. Patients displaying any of the following clinical features were recruited if their peripheral blood smears were positive for asexual forms of *P. falciparum*. Impaired consciousness was defined as prostration (generalized weakness so that the patient was unable walk or sit up without assistance) or coma (an unarousable state with a corresponding Blantyre Coma Scale (BCS) ≤ 2) where no other cause other than malaria could be identified. Multiple convulsions, defined as > 2 episodes in the 24 h period prior to admission. Respiratory distress was defined as increased work of breathing manifesting as deep, fast or very slow breathing, including retractions and the use of accessory muscles. Spontaneous bleeding was defined as physically un-induced and irrepressible bleeding from at least two non-traumatized sites in a patient with severe malaria without a previous history of abnormal bleeding. Haemoglobinuria was defined when the parent reported a history of, and the clinician macroscopically observed, dark red or black urine according to a score of 5 or more on the Hillman urine colour scale [13], a description that is more objective than a history of passing dark red or black urine alone that was previously used in the Democratic Republic of Congo (DRC) [14, 15], Nigeria [16] and Burundi [17]. Clinical jaundice was defined as the yellowing of mucous membranes noted in sufficient daylight. Pyrexia was defined as an axillary temperature of > 37.5 °C, measured using a digital thermometer and hyperpyrexia as an axillary temperature of ≥ 40.0°C. Hypoxaemia was defined as a transcutaneous oxygen saturation (TCpO_2_) of < 90%, as measured using a standard pulse oximeter. Definitions of laboratory criteria included: hypoglycaemia (blood glucose < 2.2 mmol/L), metabolic acidosis (plasma bicarbonate < 15 mmol/L), severe anaemia (Hb < 5 g/dL), hyperparasitaemia (> 5% or 250 000/μL) [8], and hyperlactataemia (lactate ≥ 5 mmol/L). On outcomes, a severe malaria death in our study was any in-hospital fatality in a child recruited to the study.

Eligible patients with any of the above features of severity were invited to participate after informed consent was sought from the patient’s parent or guardian. Data were captured on a case report form (CRF), which was logically sequenced to capture data on: socio-demographic features, clinical features (symptoms and signs), laboratory data, treatment and outcome. Blood samples for the study were collected for parasitological microscopy as well as estimation of lactate using (ARKRAY Factory, Shiga, Japan), and random blood sugar levels using On Call Plus (ACON Laboratories, San Diego, USA). In addition, a small volume of blood was collected for additional tests including 2 mls of whole blood for a complete blood count (CBC) and 0.1 mls for a quality-controlled blood slide. At the hospital, HIV testing was routinely done as part of the Ministry of Health policy.

### Malaria detection

Initial thick and thin blood smears were made from finger prick blood samples for identification and typing of *Plasmodium* parasites. Blood smears were stained with 10% Giemsa, a stable methanol based Romanowsky stain, at pH 7.2. The stained blood smears were examined microscopically under  x400 and x1000 objectives. The presence of *Plasmodium* parasites was noted based on their staining features. Malaria parasites were further quantified by examining microscopically under the x100 objective. The number of *Plasmodium* parasites in the positive smears was counted against 500 WBCs and the value that was obtained was multiplied by 16 in order to obtain the *Plasmodium* parasitaemia/μl of whole blood.

## Case management

All patient management at the PACU followed two protocols. First, the Emergency Triage and Treatment (ETAT) guidelines [18] were followed to triage all sick children on arrival into those with emergency signs, priority signs, or non-urgent cases and implementing emergency care. Second, once the emergency and priority cases had been stabilized, underlying causes for admission were treated following the Uganda National Treatment Guidelines 2010. These included but were not exclusive to antibiotic treatment for bacterial diseases such as pneumonia, meningitis, infective diarrhoea, sepsis, dysentery, and urinary tract infections. During the period of this study, anti-malarial treatment for severe malaria centred on IV quinine given intravenously at a dose of 10 mg/kg body weight in 10 mls of 5% dextrose/kg as a slow infusion over 4 h then repeating every 8 h until patients could tolerate oral medication in the same dose for a total of 7 days. Malaria presenting without features of severity was treated with the orally administered artemisinin-based combination antimalarial artemether/lumefantrine, widely marketed as Coartem^®^. When available, blood transfusion dosed at 20 mls of whole blood/kg was administered to children with severe anaemia. In addition, oxygen therapy was administered to patients with hypoxaemia. Hypoglycaemic patients were administered 25% dextrose at a dose of 2 mls/kg. Dehydration was treated with fluids according to the WHO protocols for moderate (Plan B) and severe (Plan C) dehydration. Referrals for specific care were made where appropriate, including to the Paediatric Infectious Diseases service for HIV infected children, the TB service for children with tuberculosis, the nutrition unit for malnourished children and physiotherapy for children requiring rehabilitation services. Temperature control with paracetamol and intermittent tepid sponging was administered to patients with temperatures of > 38.5 °C.

### Statistical analysis

Categorical variables were compared by use of χ^2^ tests and continuous variables using Student’s *t*-tests or ANOVA, as appropriate. Non-normally distributed variables were normalized by log_10_-transformation before analysis. P-values of < 0.05 were considered statistically significant. Variables found to be significantly associated with mortality on univariate analysis were included in logistic regression models investigating independent predictors of mortality. All analyses were conducted using R statistical software (http://www.R-project.org/).

### Ethics and patient consent

Written informed consent was provided by the parents or guardians of all study participants. Ethical permission for the study was granted by both the MRRH Research and Ethics Committee in Mbale and the Uganda National Council of Science and Technology (UNCST) in Kampala, both in Uganda.

## Results

### Socio-demographic features

A total of 10,208 children between 2 months and 12 years of age were admitted to the MRRH PACU during the period of data collection. Of these children, 6582 (64%) had a positive blood film for *P. falciparum* malaria, of which 662 (10%) had one or more clinical features of WHO-defined severe malaria and were included in the current study. Their median age was 18 (IQR 10-33) months while the majority (603/662; 91.1%) were < 5 years old (Table 2). All were residents of one of the 14 districts of the MRRH catchment area.

**Table 2:** Clinical and demographic characteristics of the study population stratified by survival status

Variable	Overall, N (%)	Survivors N (%)	Deaths N (%)	*P* value
Number	662	599	63	–
Age				
Median age (months; IQR)	18 (10–33)	18 (10–33)	18 (9–29)	0.67
Gender				
Male	382/662 (57.7)	343 (57.2)	39 (61.9)	0.48
Clinical symptoms				
Fever in this illness	648/662 (98.0)	586 (97.8)	62 (98.4)	0.76
Cough	130/662 (19.6)	121 (20.2)	9 (14.2)	0.26
Vomiting	266/662 (40.2)	242 (40.4)	24 (38.0)	0.72
Diarrhoea	238/662 (36.0)	214 (35.7)	24 (38.0)	0.71
Convulsions^b^	147/662 (22.2)	123 (20.5)	24 (38.0)	0.001
Red or cola-coloured urine	93/662 (14.0)	81 (13.5)	12 (19.0)	0.23
Clinical signs				
General				
Pyrexia (> 37.5 °C)	411/662 (62.1)	380 (63.4)	31 (49.2)	0.027
Hyperpyrexia (≥ 40.0 °C)	55/662 (8.3)	47 (7.8)	8 (12.6)	0.81
Hypothermia (< 36.0 °C)	11/662 (1.7)	10 (1.6)	1 (1.6)	0.96
Pallor	338/662 (51.1)	293 (48.9)	45 (71.4)	0.001
Clinical jaundice	177/662 (26.7)	156 (26.0)	21 (33.3)	0.21
Respiratory system				
Respiratory distress	554/662 (83.7)	501 (83.6)	53 (84.1)	0.92
Hypoxaemia (SpO_2_ < 92%)	49/662 (7.4)	35 (5.8)	14 (22.2)	< 0.0001
Cardiovascular/hydration				
Severe tachycardia^a^	291/629 (46.2)	267/570 (46.8)	24 (40.6)	0.37
Temperature gradient	251/662 (37.9)	222 (37.0)	29 (46.0)	0.16
Capillary refill time > 2 secs	135/662 (20.4)	114 (19.0)	21 (33.3)	0.007
Weak pulse	23/662 (3.5)	20 (3.3)	3 (4.7)	0.56
Sunken eyes (dehydration)	66/634 (10.4)	58/573 (10.1)	8/61 (13.1)	0.47
Abdominal				
Splenomegaly (> 2 cm)	247/662 (37.3)	216 (36.0)	31 (49.2)	0.04
Hepatomegaly (> 2 cm)	228/662 (34.4)	197 (32.8)	31 (49.2)	0.009
Neurological				
Impaired consciousness	52/662 (7.9)	43 (7.1)	9 (14.2)	0.046
Prostration	43/662 (6.5)	37 (6.1)	6 (9.5)	0.31
Coma	9/662 (1.4)	6 (1.0)	3 (4.7)	0.014

With the exception of age, figures represent numbers with column percentages in parentheses. Denominators are indicated where data are missing; splenomegaly and hepatomegaly were measured from the costal margin in the mid-clavicular line

^a^defined as > 180 beats per min in children younger than 12 months of age, > 160 beats per min in children 1 to 5 years of age, or > 140 beats per minute in children older than 5 years of age

^b^ > 2 in 24 h; P-values between survivors and deaths were estimated by χ^2^ tests with the exception of age, which was compared by the Kruskal–Wallis test

### Clinical features

**Fig. 1:**
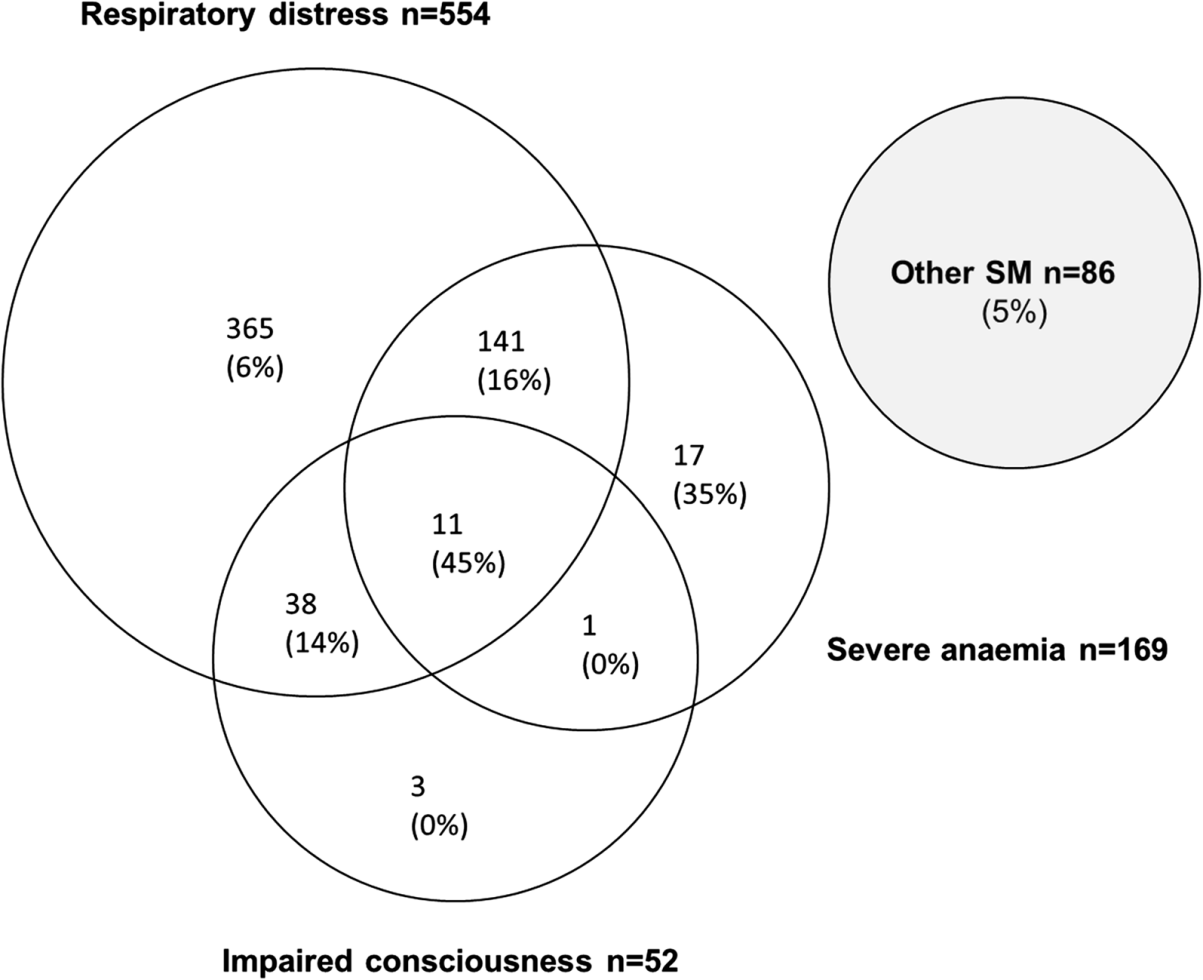
Figures indicate the number of cases with features of each clinical phenotype or phenotype combination, with the case-fatality rate in parentheses. Other SM was defined as the presence of any WHO-defined feature of severe malaria in the absence of respiratory distress, severe anaemia or impaired consciousness

The clinical and laboratory characteristics of recruited children, stratified by survival to hospital discharge, are summarized in Table 2. Respiratory distress was observed in a high proportion (83.7%) but was particularly common in those < 5 years old (92.6%). A lower proportion (7.9%) showed signs of impaired consciousness while strictly defined cerebral malaria was rare (1.4%). Decorticate, decerebrate or opisthotonic posturing was not observed in any of the cases. Overall, both clinical pallor (338; 51.1%) and clinical jaundice (177; 26.7%) were common. The recent passage of dark red or black urine was reported in 14.0%, of whom a high proportion also had either clinical jaundice (74.2%) or severe anaemia (43.0%). Signs of impaired perfusion were seen in a high proportion: severe tachycardia in 53.7%, a temperature gradient in 37.9% and a prolonged capillary refill time (CRT) in 20.4% (Table 2). More than one clinical or laboratory feature of severe malaria was seen in a high proportion of cases (Fig. 1).

### Laboratory characteristics

The laboratory characteristics of recruited children are summarised in Table 3. Although parasite densities were high overall (geometric mean 58 800; 95% CI 49 700–69 600 parasites/µL), densities of < 500 parasites/µL were seen in a small proportion (1.0%). Severe anaemia was present in 25.5% of patients, most of whom were < 5 years of age. A high proportion of severely anaemic children also manifested other clinical signs of severe malaria, including respiratory distress (89.3%) and hyperlactataemia (40.2%). Only 3.6% of children were hypoglycaemic.

**Table 3:** Laboratory parameters among patients, stratified by survival status

Laboratory parameter	Overall, N (%)	Survivors (%)	Deaths (%)	P-value
Number	662	599	63	n/a
Parasitaemia				
Geometric mean (parasites/μL; 95% CI)^a^	58 800 (49 700–69 600)	61 110 (51 100–73 000)	41 390 (25 060–68 300)	0.078
Hyperparasitaemia^b^	365/585 (62.4)	331/558 (62.6)	34/57 (59.6)	0.65
Biochemistry				
Median Lactate (mmol/L; IQR)	2.2 (1.7–4.0)	2.1 (1.7–3.6)	5.6 (2.6–9.3)	0.004
Hyperlactataemia (≥ 5 mmol/L)	134/660 (20.3)	100/597 (16.6)	34/63 (53.9)	<0.0001
Median blood glucose (mmol/L; IQR)	6.8 (5.5–8.4)	6.8 (5.5–8.4)	7.4 (5.2–10.0)	0.24
Hypoglycaemia (< 2.2 mmol/L)	21/577 (3.6)	15/527 (2.8)	6/50 (12.0)	0.004
Haematology				
Median Haemoglobin (g/dL; IQR)	7.1 (4.9-10.1)	8.0 (5.1-10.1)	4.8 (3.3-9.7)	<0.0001
Severe anaemia (Hb < 5 g/dL)	169/662 (25.5)	136 (22.7)	33 (52.3)	<0.0001
Leucocytosis^c^	176/632 (27.8)	152/571(26.6)	24/61 (39.3)	0.035
Thrombocytopaenia^d^	263/632 (41.6)	237/571 (41.5)	26/61 (42.6)	0.87

Figures show N’s with proportions in parentheses

^a^n = 585, *CI* confidence interval, *IQR* interquartile range; P-values were estimated by χ^2^ tests for categorical and Student’s *t*-tests for continuous variables

^b^as defined in Table 1

^c^white blood cell count > 11x10^3^ cells/mm^3^

^d^platelet count < 150x10^3^/μL

### Outcome

Overall mortality was 9.5%, but this varied by clinical phenotype as summarized in Table 2 and Fig. 1. For example, although mortality was only 9.6% in children with respiratory distress, it was considerably higher (33.3%) in children with cerebral malaria. Similarly, mortality also varied in children categorized by a range of laboratory characteristics (Table 3). The case fatality rate (CFR)–the proportion of children who died in hospital–was 19.5% in children with severe anaemia and as a consequence this complication was a major contributor to overall mortality (33/63; 52.4%). As median lactate was significantly higher in children who died (5.6 mmol/L; IQR 2.6–9.4) than in those who survived (2.1; 1.7–3.6) (*P *= 0.004), hyperlactataemia was strongly associated with mortality (P < 0.0001). Hypoglycaemia was also associated with mortality (*P *< 0.001), but no correlation was found between admission parasite density and outcome. In general, CFRs increased with the number of severity features reported (Fig. 1). While a number of clinical and laboratory features were associated with outcome on univariate analysis (Tables 2 and 3), the only features that remained significant on multivariate analysis were severe anaemia (*P *= 0.0002), hypoxaemia (*P *= 0.008), hyperlactataemia (*P *= 0.001) and hepatomegaly (*P *= 0.004) (Table 4).

**Table 4:** Logistic regression analysis for predictors of mortality

Variable	Univariate logistic regression		Multivariate logistic regression	
	Odds ratio (95%CI)	*P*-*value*	Odds ratio (95%CI)	*P*-*value*
Clinical				
Pyrexia	0.56 (0.33–0.94)	0.027	0.79 (0.40–1.57)	0.50
Pallor	2.61 (1.48–4.61)	0.001	0.84 (0.34–2.12)	0.98
Coma	4.94 (1.20–20.2)	0.01	1.52 (0.28–8.23)	0.62
Delayed capillary refill	2.13 (1.21–3.73)	0.007	0.82 (0.35–1.93)	0.64
Splenomegaly	1.72 (1.02–2.89)	0.040	1.00 (0.46–2.18)	0.75
Hepatomegaly	1.98 (1.17–3.33)	0.009	2.29 (1.29–4.06)	0.004
Hypoxaemia	4.60 (2.32–9.13)	1.25 × 10^−5^	3.64 (1.39–9.52)	0.008
Laboratory				
Severe anaemia	3.74 (2.20–6.36)	1.05 × 10^−6^	5.36 (2.16–1.32)	0.0002
Hyperlactataemia	5.83 (3.40–9.50)	1.58 × 10^−7^	3.66 (1.72–7.80)	0.001
Hypoglycaemia	3.84 (1.44–10.19)	0.004	2.25 (0.63–7.98)	0.21
Leucocytosis	1.79 (1.04–3.09)	0.035	1.03 (0.49–2.14)	0.93

## Discussion

To the best of our knowledge, this study is the first to describe the clinical and laboratory features of childhood severe *P. falciparum* malaria in Eastern Uganda that follows the standard WHO criteria [12]. Moreover, the sample size was large in comparison with similar earlier studies conducted elsewhere [4, 5, 19] and to a recent study conducted in Kampala, Central Uganda [20]. The median age of the children recruited was 18 months and the majority were < 5 years old. The common clinical features recorded were respiratory distress, clinical jaundice, severe anaemia, hyperlactataemia and the recent passage of dark red or black urine. The overall case fatality rate was high (9.5%) and increased with the number of clinical manifestations recorded.

In the current case-series, 14% of children reported a history of having recently passed dark red or black urine, consistent with the diagnosis of haemoglobinuria or blackwater fever. This figure is close to the 14.5% previously reported among children recruited to the FEAST trial at the MRRH site [7]. While the entry criteria for recruitment to that study—severe febrile illness with impaired perfusion—were different from those in the current study, the similarly high prevalence confirms the current importance of this condition in Eastern Uganda. Although historically, the passage of dark urine has not been reported frequently among children, a growing number of case-series have been published in recent years that have described this problem in both Africa [14–16, 21, 22] and Oceania [23]. Many previous reports have been based on the subjective reporting of urine colour by patients, parents or guardians, as recommended by the WHO. In this study, however, in addition to such reports urine colour was also corroborated by asking the parents to match the colour of their child’s urine to the corresponding colour on the Hillmen urine colour scale [13]. Additionally, samples collected after admission were verified against this scale by attending clinicians. While the lack of systematic urine testing is a weakness of the study, the use of the colour scale adds to the veracity of the reported data. Unlike descriptions from Nigeria [16] and the Democratic Republic of Congo [22], none of the children presenting with dark urine in the current study gave a history suggestive of acute renal failure, although subclinical renal impairment cannot be excluded on the basis of clinical history alone. Both the aetiology of dark urine and the reasons for its more recent increase among children with malaria in Africa are incompletely understood, but we have previously suggested that it might be related to the recent policy change towards the use of artemisinin-based combination therapy (ACT) as first-line treatments of malaria [7]. Further work examining this subject is ongoing.

Notable was the high proportion of children who presented with respiratory distress. Respiratory distress in children with severe malaria can be categorized into two major components: deep breathing, which involves an abnormally increased amplitude of chest excursion, and indrawing (or nasal flaring in younger children), often associated with an increased rate of breathing [24]. Deep breathing has been found to correlate closely with the presence of acidosis in previous studies [25] while chest indrawing is often a sign of lung parenchymal disease, including pneumonia [24]. Both components are included in the WHO definitions of severe malaria [12, 24]. In the current study, data regarding the individual components of respiratory distress were not recorded separately, but the fact that the correlation between respiratory distress and acidosis was low suggests that parenchymal disease may have been present in a high proportion of recruited cases. Previous studies have suggested that lung disease is a less common manifestation of severe malaria than deep breathing [25], raising the possibility that malaria may not have been the primary cause of severity in a proportion of children recruited to the current study, highlighting the difficulties in phenotyping children based on clinical evidence alone.

In keeping with the local epidemiology of heavy perennial malaria transmission [26], severe malarial anaemia was significantly more common than cerebral malaria in the current case-series. The spectrum differed markedly from that reported from areas of lower transmission, where neurological manifestations in older patients predominate [2, 3]. For example, in one study, conducted in Kampala, Uganda, children with cerebral malaria were significantly older (median 2.5 years, IQR = 1.5–3.9 years) than children without cerebral malaria (1.7 years, IQR = 1.0–2.9 years; *P *< 0.0001) [20]. The case fatality rate in cerebral malaria is generally high with studies typically reporting rates that range between 10 and 40% [2, 27, 28]. In the AQUAMAT trial [29], even in children who were treated with artesunate mortality exceeded 20% in those with profound coma (BCS ≤ 2), a figure that is broadly consistent with that seen in our current study. Although not measured in this study, long-term neurological sequelae are common in children admitted with cerebral malaria [30], potentially resulting from recurrent convulsions, hypoglycaemia and acidosis [2, 6].

In contrast to the recent mortality rates reported from the TRACT trial [31, 32], which examined blood transfusion strategies in multiple settings (including two centres from Eastern Uganda), inpatient mortality among children presenting with severe malarial anaemia in the current observational study was high (19.5%). The lower mortality seen in TRACT might have reflected the fact that recruitment to the trial was halted when blood for transfusion was not available or because of improvements in blood transfusion services in Uganda during the intercurrent period. In the current study, the receipt of blood transfusions was not recorded; however, a high rate of early mortality among critically sick children with severe anaemia while awaiting a blood transfusion has been previously described [33]. The importance of adhering to the restrictive blood transfusion policy from the WHO was underpinned by evidence from children with severe and uncomplicated anaemia within TRACT [31], who did not require immediate transfusion, allowing donor blood to be targeted to those with severe and complicated anaemia in situations where supplies are limited.

In conclusion the common clinical features of severe *P. falciparum* malaria among children in the current case-series were respiratory distress, clinical jaundice and severe anaemia. Clinical jaundice and the recent passage of dark red or black urine, neither of which have been commonly reported in similar studies from other parts of Africa, were frequent. Overall mortality was high, with severe anaemia, hyperlactataemia, hypoxaemia and hepatomegaly being independently associated with death. Observations from this study justify further work aimed at investigating the causes and best treatment for blackwater fever in children in Eastern Uganda.

## Data Availability

The study data are available by request to the corresponding author.
